# Climax thinking on the coast: a focus group priming experiment with coastal property owners about climate adaptation

**DOI:** 10.1007/s00267-022-01676-x

**Published:** 2022-06-30

**Authors:** Kate Sherren, Krysta Sutton, Ellen Chappell

**Affiliations:** School for Resource and Environmental Studies, Dalhousie University, Halifax, NS Canada

**Keywords:** Climate change, Coastal adaptation, Environmental communication, Experimental treatment, Framing, Nature-based solutions

## Abstract

Coastal communities face increasingly difficult decisions about responses to climate change. Armoring and defending the coast are being revealed as ineffective in terms of outcomes and cost, particularly in rural areas. Nature-based options include approaches that make space for coastal dynamism (e.g., through managed retreat) or leverage ecosystem services such as erosion control (e.g., by restoring coastal wetlands). Resistance can be strong to these alternatives to hard infrastructure. Nova Scotia, off Canada’s Atlantic coast, is a vulnerable coastal jurisdiction facing such decisions. The emerging climax thinking framework was used to design 14 experimental online focus groups. These focus groups explored how three priming treatments influenced discussions about adaptation options and urgency and quantitative pre/post-tests, compared with information-only control treatments. A future-focused priming strategy seemed most effective since it fostered discussions about duties to future generations. The altruism-focused priming strategy involved reflections of wartime mobilization and more recent collective action. It also worked but was more difficult to implement and potentially higher risk. Past-focused priming was counterproductive. Further research should test the future-focused and altruism-focused strategies among larger groups and in different jurisdictions, reducing some of the biases in our sample.

## Introduction

There is nowhere on Earth that is untouched by climate change, but it is arguably on coasts—so often densely populated—that the impacts are felt first and most dramatically (He and Silliman 2019). Changing the *way* we solve climate challenges calls for changing land covers and land uses, and landscape transformation rather than incremental change (Pelling et al. 2015; Elmqvist et al. 2019). Options other than vigorous defense of existing coastal uses are hotly contested and politically fraught (Gibbs 2016), particularly in the Global North, which bears more responsibility for climate change *per capita* (Hickel 2020). Nature-based coastal adaptation options go by many names. As a category, they encompass approaches that allow the realities of coastal processes to align with human expectations and needs, typically working from ecological principles and within socio-political realities to moderate coastal climate change impacts (Van Proosdij et al. 2021). Included are “living shorelines” restoration work to enhance and maintain natural coastal processes, but also accommodation and retreat, which call for a recalibration of what makes for a good coastal life and of our capacity for control in coastal ecosystems (Mach and Siders 2021). Nature-based coastal adaptation options often require significant lead-time to implement and become effective, which means that coastal residents may be asked to change before full impacts are felt. Prospect theory tells us that decision-making in such a state of uncertainty will typically lead to conservatism (Kahneman 2011).

Climate researchers and practitioners of mitigation and adaptation options have long explored how to communicate with the public about the nature and scale of change required (Nerlich et al. 2010). *Framing* refers to designing communications by highlighting elements that will resonate best with target audiences. *Priming*, by contrast, sets the stage for target audiences to make desirable associations with messages instead of undesirable ones (Chong and Druckman 2012). Where framing integrates the desired interpretation with the message, priming designs the context in which messages are received, for instance, what was discussed immediately before, so compatible messages are more accessible to the audience than incompatible. A systematic literature review found most climate change framing research was observational (e.g., news media) and explored scientific, economic, and environmental dimensions; experimental designs and frames such as morality/ethics were rarer (Badullovich et al. 2020). Framing experiments are often criticized for using convenience rather than real target samples (Chong and Druckman 2012). A recent Delphi study with climate practitioners in the United States found that the effectiveness of framings focused on future generations or on current generations was not determined by political allegiance (Stern et al. 2020). An international conjoint study found that framing focused on environmental and health impacts of climate change led to more support for climate change policy and those focusing on migration impacts led to less, with similar patterns seen for framing of global impacts over personal, and short-term impacts over long-term (Dasandi et al. 2021). Comparatively, little research exists testing priming effects in the climate context, and what does exist usually involves large-scale surveys (Schuldt and Roh 2014; Lutzke et al. 2019). Some priming research, however, suggests that moral frames can be useful when targeting challenging audiences for climate messages (Zaval et al. 2015; Schuldt et al. 2017). This research was designed to help us understand how to foster productive discussions with coastal residents about nature-based coastal adaptation. We deployed experimental focus groups including pre- and post-tests on the coast of Nova Scotia, Canada, an area highly exposed to climate impacts. Our purpose was to compare three experimental priming treatments with information-only control treatments using mixed methods: (1) pre/post measurements of climax thinking, and (2) focus group sense of urgency around climate adaptation, and openness to nature-based options, including retreat. This paper first explains the climax thinking framework used as the basis of the priming treatments, then describes the focus group experimental design and the pre- and post-tests that serve as our primary datasets. We then present statistical and then qualitative analysis of our preliminary operational measure of climax thinking and how it was influenced by the focus group treatments. The discussion explores the findings in the context of the literature and makes recommendations for further work.

## Background

### Climax Thinking

By contrast with relict “cultural” landscapes, our everyday lived landscapes are relatively mundane. However, they represent significant planning challenges (Plieninger et al. 2015), in part because of the subjectivity in how they are experienced and interpreted (Stedman 2016). Narrow sets of landscape options become dominant and normalized through time and as power dynamics play out (Stokowski 2002; Siders and Keenan 2020). Dominant landscape ideals represent a powerful sociological imaginary (Ingold 2012): what we collectively hold as possible and desirable is often limited by what already exists. In a recent review, the term *lock-in* was used to describe the various forms and conceptions of “undesirable resilience” that limit needed transformation (Dornelles et al. 2020). Climax thinking is an emerging framework for understanding such undesirable resilience to public good landscape change that mirrors succession theory in plant ecology.

Climax thinking is the widespread misconception, characteristic of Western contexts, that our current landscapes are ideal or even fated (Sherren 2021). It uses the metaphor of succession theory in plant ecology, developed in rangelands (Sayre 2017), where a climax plant community was seen as a stable one that dominates in a given site and set of conditions after a predictable sequence of previous communities. Early ecologists believed this equilibrium state was inevitable, and that it would be reliably returned to after disturbance if properly managed. In the West, we seem to similarly perceive our lived landscapes as progressing from “pioneers” to what is seen locally as a mature or climax state, one that must be returned to after disturbances like floods. But non-equilibrium concepts, such as panarchy and resilience, now dominate ecology, acknowledging the potential for multiple stable states for any given social ecological system (Briske et al. 2020). Social scientists tend toward more positive notions of climax (i.e., preoccupations with stable places and attachments (Brown et al. 2020)), despite the fact that it is often economic power that allows us in the West to delegate responsibility for maintaining the *status quo* to those more geographically or psychologically distant. Climax thinking has been deployed to help understand perceptions of wind energy, where it was found that even turbines can become part of that accepted “climax” state (Chappell et al. 2020).

The original description of climax thinking presents alternate ways the hypothesized drivers of ignorance and exceptionalism might attenuate concern over time and space (Table 1 rows 1 and 2; Sherren 2021). The *past dimension* asks if we are ignorant of former land uses or dismiss the impact of changes on earlier occupants because we can’t relate to them. The *future dimension* asks if we think future generations matter less than ours or merely assume that our current landscape solutions will continue to meet future needs. The *space dimension* describes self-other dynamics. For instance, do we believe we lack the ability to adapt to landscape change or that we should not have to? Alternatively, do we think that holding local landscapes in stasis has no impacts elsewhere, or that people elsewhere simply matter less? In this paper, climax thinking is used in two ways. First, we measure the various dimensions of climax thinking quantitatively in pre- and post-tests around the focus groups. Second, the priming treatments each leverages one of the three climax thinking dimensions: past, future, or self-other (called *altruism* in this paper, as will be explained).

### Public Perceptions of Nature-based Coastal Adaptation

Despite climate-related risks, coastal residents typically resist precautionary coastal retreat or changes from the “hard” infrastructure they know and trust to natural protective infrastructure (Matthews et al. 2015; Gibbs 2016; Mallette et al. 2021). Where disasters have already occurred or the risk of one is perceived as unacceptable, coasts and communities must often be managed in new ways (Siders et al. 2019; Buma and Schultz 2020). Managed retreat or accommodation (adapted infrastructure and land use) have been underway on many soft or low coasts (Milligan and O’riordan 2007; Neuvel and Van Der knaap 2010). European de-polderization, for instance, attracts quite mixed opinions (Goeldner-Gianella 2007). Coastal retreat via buyouts has been increasingly implemented in the United States (Rosenzweig and Solecki, 2014), albeit with some concerns about justice issues (Siders and Keenan 2020). While there is some work on government relocation in Canada (Loo 2019), there is not yet much research on climate-related relocation experiences (Woodhall-Melnik and Weissman 2021).

By contrast with retreat and accommodation, which promise a change in the use of existing land, living shorelines options typically involve changing coastal and offshore habitats to act as a natural buffer (Smith et al. 2020). Cooper and Pile (2014) would characterize such options as *resistance* rather than adaptation: the latter changes human activities to allow the environment to change, and the former resists environmental change so humans do not need to adapt. Living shorelines are not, of course, a panacea for coastal climate change (Seddon et al. 2020); while many people like the look of natural defenses, they often trust the engineered ones more (Gray et al. 2017). Most of the research on landowner perceptions of living shorelines, such as salt marshes and constructed reefs, has been done in the northeastern United States. For instance, researchers have used surveys, shoreline damage data, and/or shoreline modification permits to understand decisions to armor or use living shorelines on the Eastern Seaboard (Scyphers et al. 2015; Stafford 2020), where hard defenses often do not meet owner expectations (Smith et al. 2017). Appreciation of constructed and restored coastal wetlands, which are often used as elements of living shorelines, can be difficult because of their flatness and relative invisibility (Yamashita 2014). On the Bay of Fundy coast of Nova Scotia, remnant tidal wetlands have been shown to be largely “overlooked”: seen but not recognized and rarely advocated for (Sherren et al. 2016; Chen et al. 2020). Public knowledge and appreciation of the elements that compose living shorelines options are critical to acceptance.

## Methods

We integrated mixed methods (*sensu* Walker and Baxter 2019) to explore the impact of communicative priming devices based on climax thinking upon conversations with coastal residents about nature-based coastal adaptation options. This approach combined the richness of focus groups with the precision of quantitative surveys, providing opportunities to triangulate across methodological lenses. Online delivery reduced barriers to participation among a widely distributed rural population.

### Study Area

Atlantic Canada will likely struggle more than Canada’s other coasts to manage climate change (Vasseur et al. 2017): flooding will be worse, relative sea level rise will be higher, and projected increases in frequency and severity of storms will bring storm surges and overland flooding (Savard et al. 2016). The region has a disproportionately high concentration of high-risk flood areas (Thistlethwaite et al. 2018) and older demographics (Manuel et al. 2015), and it economically depends on functioning coasts for resource exploitation, tourism and trade (Rapaport et al. 2017). Atlantic coasts are also overwhelmingly rural, meaning ongoing “hard” protection measures are often not economical (Danielson 2019; Henstra et al. 2019). Much Canadian adaptation research has focused on Central or Western contexts (Morrison et al. 2018), often urban ones (Haney 2019; Doberstein et al. 2020). The urban Atlantic coast in the United States is also well studied thanks to recent storms (e.g., Siders and Keenan 2020). Yet insights do not always transfer easily between jurisdictions (Bogdan et al. 2022).

Nova Scotia is a small and highly coastal province within the Atlantic region of Canada, comprising a peninsula joined to the rest of Canada through a narrow isthmus, one large island (Cape Breton), and thousands of smaller islands. The furthest distance to the coast in the province is 67 km, but the coastal morphology varies widely. The Bay of Fundy coast at the end of the Gulf of Maine is muddy and marshy, the Northumberland Strait coast facing Prince Edward Island features highly erosive bluffs, and the Atlantic coast has more complex rocky shorelines (Savard et al. 2016). Each of the province’s 13 coastal ecosystem types is affected in different ways by climate change. In particular, the sea level rise that all of Canada is experiencing is more severe in relative terms in Nova Scotia because the province is simultaneously sinking geologically as a response to glacial retreat and geological springback in Canada’s center (Savard et al. 2016). Being a coastal culture, as well as relatively long settled by North American standards, Nova Scotia coasts host significant built infrastructure and are predominantly privately owned, leading to persistent development pressure (Rapaport et al. 2017). One of the more unusual types of coastal infrastructure in the region are dyked and drained agricultural lands created from salt marshes. These were created by early French settlers, called Acadians, starting in the 1600s. These dykes and protected dykelands face particular challenges from sea-level rise (Sherren et al. 2019).

### Online Focus Groups

We used online focus groups because of the difficulty of bringing coastal residents together over such large distances. Online focus groups were somewhat new to scholarly research during this pre-Covid work, though they had been commonly used in market research (Stewart and Shamdasani 2017). Such focus groups are implemented using a conference call system, where participants use a desktop or laptop computer to view interactive material, such as videos and short polls, but receive their audio through a phone. The calls were targeted at 75 min each, though several ran to 90 min. Participants were all compensated $75 for their time to participate, irrespective of the contributions they offered. The focus groups included five to eight participants each. National polling firm Narrative Research Inc. recruited participants by using landline phone calls and professionally facilitated the focus groups.

We selected focus group participants who owned property within 5 km of each of the three morphologically distinct coasts described above (Bay of Fundy, Northumberland, Atlantic), anticipating that they may have different experiences and preferences to discuss. Limiting focus groups by coast also allowed us to present only adaptation options relevant to each specific coast. Four focus groups were recruited in each of the Atlantic and Northumberland coasts, one per planned treatment (see below). Eight focus groups (two replications of each priming treatment) were planned in the Bay of Fundy area because it is also the focus of other projects, but recruitment challenges meant we could only run six. Therefore, only two treatments received replications there. All the focus groups took place between June and early August 2019. They occurred by coast: first Atlantic, then Northumberland, and finally Bay of Fundy.

The focus groups included three video components as well as numerous discussions and specific quantitative polls used only to stimulate conversations (i.e., polls were not analyzed statistically). All focus groups began with a set of instructions (how to digitally “raise hand”) and ethics disclosures (e.g., treatment of data, that researchers were listening in). This was followed by participant introductions (first name only), during which participants were asked to describe the nature of the coast where they lived (e.g., rocky, sandy, bluffs, etc.). Then all participants watched the same 5-minute video that introduced the impacts of climate change across the Nova Scotia coast. This was designed to develop a shared understanding about climate impacts. A poll and discussion followed, during which participants reported on and described their local experiences of climate change impacts, particularly those related to rising sea levels. Next, all participants watched the same 2-minute video that provided some vocabulary for talking about coastal protection options, including hard versus soft protection, accommodation, and retreat. This helped us to understand public perceptions of these options to support our work in the region (Sutton 2020). A discussion and poll followed about what coastal protections they are seeing in their areas and how effective they seem. At this point in the focus group, the facilitator would apply the specific treatment that was being tested.

Three groups began facilitated discussions (treatments) about a topic that was conveyed as separate from the previous focus of sea-level rise. Each topic covered one dimension of climax thinking, specifically what topic might reduce that dimension of climax thinking if used to prime conversations of adaptation.

- *Past priming* sought to convey to participants that the dramatic changes needed for coastal adaptation are only the most recent in a long line of changes people have faced on the same coast as their needs have changed (Table 1, row 3). This was implemented by a facilitated discussion of past changes on their coast, for instance due to changing economic base, and how citizens coped (Table 1, row 4). Such a discussion was designed to counter unreflective (or perhaps selective) nostalgia, as described by Lowenthal (1975), and to remind participants of the longer term anthropogenic trajectory (Hanley et al. 2009).

**Table 1:** A simplified pathology of climax thinking and how it was implemented in the focus groups

	Past	Future	Altruism
Pathology- Exceptionalism	Previous land uses were just paving the way for this one	Future generations matter less than this one	Someone else should need to accept change before I do.
Pathology-Uncertainty	There were no previous land uses	Current solutions will continue to work in future	Local landscape decisions do not affect people elsewhere
Focus group priming	This change is just one of many your coast has faced over time as needs change.	The things you love about being on the coast will persist under adaptation.	We have faced big challenges together before and can do so again.
Specific discussion topics	How has your coast changed for reasons other than climate change (e.g., economy) and how did the community cope?	What do you love about this coast that you hope future generations will get to experience, and what is your duty to those future residents?	How did the residents of your community face wartime mobilization and what made that possible?
- *Future priming* sought to explore the idea that adaptation would not erase their enjoyment of living on the coast. This was achieved through a discussion about what participants enjoyed about living on the coast that they would also like future generations to experience (e.g., sound, smell, view, community), and whether they might need to act to ensure that future enjoyment. This aligns with other effective future-oriented framings described in the climate literature (Zaval et al. 2015; Stern et al. 2020).
- *Altruism priming* sought to explore the benefits of associating sacrifices in the name of climate change with other satisfactions of collective action, inspired by Solnit (2010). This priming experiment used wartime mobilization as a point of discussion, despite some debates in doing so (Kester and Sovacool 2017; Patterson et al. 2021), asking what sacrifices their communities made to help the war effort and whether they work together today in any way. While wartime generations are fading, even the smallest towns have a cenotaph to commemorate the region’s involvement with past wars, and the sacrifices of war are remembered every November 11^th^ (Remembrance Day); we expected such stories would also be passed down within families.

Facilitators presented treatment-specific poll questions to each focus group, which are not explored here as they were used simply to generate discussion. In order to prime, we used facilitated discussion, rather than another video or other “canned” message, because we believed it would be easier and more authentic for participants to tell locally relevant stories to one another than for non-local researchers to do so. Groups assigned to the control treatment had none of the above discussions and transitioned straight to the nature-based adaptation discussion below.

The final component of all focus groups was a 6–7-min video about viable “nature-based” options (excluding the hard options described earlier such as seawalls). All the focus groups received information about overland flow management, living shorelines (natural bank stabilization, dune building, wetland buffer, oyster reefs and reef balls, and beach nourishment), accommodation (raising homes or infrastructure, changing production systems), and retreat (moving things back and/or up to leave space for dynamic coastal action). The most time was spent on living shorelines, because it is not a single strategy but a range of different options. Only Bay of Fundy participants were presented with the option of dyke realignment (straightening and/or pulling dykes back to provide space for wetland restoration), as that is the only shore where agricultural dykelands are common (Ollerhead et al. 2020). After each of the options was described, participants did a poll about whether they supported its use on their coast. At the end of this section, participants responded to a poll about their *preferred* solution. The subsequent discussion explored perceptions of these nature-based options, what coastal residents would need to know before using/accepting them, and how to talk to coastal residents about them. The final question asked what participants thought about their priming discussion topic in the context of a climate change discussion and was the only one that explicitly connected the priming discussion to climate adaptation (though some participants did that themselves). The focus group closed with thanks and further instructions on receiving their incentives.

Focus groups were recorded, transcribed using InqScribe, and analyzed to understand the conversations during each priming treatment (see Supplementary Materials 1) and about coastal adaptation options. NVivo 12.0 was used to organize and code the data, using a general inductive approach as opposed to searching for established themes (Thomas 2006). It is important to note that not all participants answered each question, because of time constraints, so the unit of analysis here is the focus group (specifically the coast and treatment) rather than the individual. Matrix queries were used to separate the induced themes by treatment.

### Pre- and Post-tests

Short survey-based pre- and post-tests were run with participants to provide a test of climax thinking and its mobility because of the focus group treatments. The pre-test was done at the time of recruitment when other demographics were captured, generally within a week of the focus group, and the post-test within hours or days of finishing the focus group. As the two surveys were implemented quite close to one another, two similar but not identical question sets were prepared. The surveys included eight statements for Likert response, which were designed as a first operationalization of the theorized dimensions of climax thinking discussed in the Background, including cause (exceptionalism, ignorance) and both temporal and spatial dimensions (Table 2). Principal Components Analysis (PCA), Cronbach’s alpha, and Spearman tests assisted us in establishing scales for the preliminary comparison of pre- and post-tests. Beyond descriptive statistics, we use paired and unpaired t-tests and Spearman for bivariate analysis.

**Table 2:** Descriptive statistics for pre- and post-test statement Likert scores, % completely disagree to completely agree

Climax dimension	Pre-test (*n* = 97)	Pre (%) Mean (SD)	Post test (*n* = 81)	Post (%) Mean (SD)	Pre-post t-test
Past - E	1a. I feel grateful to those who worked to make this coast what it is today.	3, 3, 27, 33, 34 **3.92** (1.01)	2a. Mine is the kind of coastal landscape that previous residents were working towards.	10, 21, 42, 17, 10 **2.96** (1.10)	5.39***
Past -I	1b. I think the coast has always looked pretty much the same way that it does now.	33, 34, 12, 19, 2 **2.23** (1.16)	2b. It to be living in a place that has changed so little over the years.	9, 20, 23, 33, 15 **3.26** (1.19)	−6.47***
Future-E	1c. Decisions about the coast must consider the needs of its current residents above future residents.	13, 29, 22, 22, 14 **2.23** (1.16)	2c. Future generations will have their own opportunity to make decisions about the coast; this is our turn.	15, 22, 12, 36, 15 **3.14** (1.33)	−0.98
Future-I	1d. Our present coastal protection options have served us well and will protect future generations as well.	14, 40, 31, 12, 2 **2.47** (0.96)	2d. Future coastal protection options will be pretty much the same ones that we have today.	15, 27, 17, 36, 5 **2.89** (1.19)	−1.91
Self-E	1e. Decision-makers must do whatever is necessary to maintain my coastal landscape.	2, 10, 20, 42, 26 **3.79** (1.01)	2e. I should not need to be personally affected by changing coastal conditions.	32, 31, 20, 12, 5 **2.27** (1.18)	9.07***
Self-I	1f. I could not cope with having significant changes to my cherished coastal landscape.	6, 15, 32, 32, 14 **3.33** (1.10)	2f. I could never get used to significant changes in the coastal landscape at this stage in my life.	23, 31, 17, 21, 7 **2.58** (1.26)	4.35***
Other-E	1g. People should deal with their own coastline before worrying about others’.	24, 33, 26, 15, 2 **2.39** (10.08)	2g. Our community is more deserving of public support to maintain its coastline than some others.	19, 26, 33, 10, 12 **2.72** (1.24)	−1.33
Other-I	1h. What I do on my coast is nobody’s business but my own.	38, 41, 10, 8, 2 **1.95** (1.00)	2h. Coastal management decisions made in one place have no impact elsewhere.	41, 38, 5, 12, 4 **2.00** (1.14)	−0.44

Climax dimension refers to dynamic (temporal [Past/Future] or spatial [Self/Other]) and pathology (Exceptionalism, Ignorance)

***p < 0.001

## Results

### Participant Demographics

There was a total of 99 focus group participants: 27 from the Atlantic coast, 25 from the Northumberland coast, and 47 from the Bay of Fundy coast. The Bay of Fundy coast had two additional focus groups, six instead of four. The experimental treatments were applied to uneven numbers of participants, in part because of the unpredictability of who shows up for focus groups after recruitment and in part because of the extra future and altruism focus groups run in the Bay of Fundy. The past treatment was applied to 17 participants, future to 26 participants, altruism to 30 participants, and control to 23 participants.

Participants were largely female (56%), owners or co-owners of direct ocean frontage (73%), and year-round, rather than seasonal, residents (85%). Most participants were parents (86%), though given the age distribution of participants (70% were 55+, another 21% were aged 45–54), it is likely many of those children no longer lived at home. Indeed, most reported living in two-person households (65% had two-person households, 11% one-person, 24% three-person or more). This oversamples older demographics by about double: those 55 and over only compose a third of the population in rural Nova Scotia and small population centers (Statistics Canada 2011). The older demographic captured was likely the result of recruitment by landline telephone, which older people are most likely to use and to answer when called by unfamiliar numbers. However, it should be noted that the older demographic is also more likely to own coastal property.

Other variables captured about residents during recruitment were more diverse. We required that participants had been coastal residents for at least five years, but a quarter of participants said they had been resident for 51 years or more. Education included high school or less (20%), college (38%), and university (41%) experience, and three-quarters of reported salaries were between $45,000 to 100,000 per annum (with 14% falling below this range and 9% above it). Many participants were retired (44%), but many also worked full time (28%) or were self-employed (16%); of the remaining participants, 10% were partially employed (part time or seasonal) and only 2% were not employed outside their home (one homemaker, one unemployed).

### Measuring Climax Thinking

The two surveys (pre- and post-tests) were completed by 95 and 84 participants, respectively. Mean pre-test results tended toward agreement on three statements: the one designed to test past-oriented exceptionalism (“I feel grateful to those who worked to make this coast what it is today”) and both self-oriented statements (“Decision-makers must do whatever is necessary to maintain my coastal landscape” and “I could not cope with having significant changes to my cherished coastal landscape”) (Table 2). The rest of the pre-test statements garnered general disagreement, but particularly the statement designed to test ignorance about impacts on others (“What I do on my coast is nobody’s business but my own”).

The statement designed to test ignorance about impacts on others received the most disagreement in the post-test as well as the pre-test, though it was worded differently: “Coastal management decisions made in one place have no impact elsewhere.” Unlike in the pre-test, where it was largely agreed with, the self-oriented exceptionalism statement was largely disagreed with post-test (“I should not need to be personally affected by changing coastal conditions”). Most other statements hovered around neutral in the post-test. Different statements garnered the most agreement in the post-test, however: “It feels good to be living in a place that has changed so little over the years” and “Future generations will have their own opportunity to make decisions about the coast; this is our turn.”

Statistics suggest that both of the eight-statement sets are borderline acceptable for use as a novel scale (Hair et al. 2014), with alphas between 0.69 and 0.70 for the full pre/post set and for pre- and post-tests separately (Table 3). Responses to these statements are averaged for use as Climax-Pre and Climax-Post scales. Intended scales along dimensions such as past, future, other, exceptionalism, and ignorance (Table 3) were not consistently strong enough to use as scales, but it is not a unidimensional dataset. Principal Component Analysis with Varimax rotation was used to explore the dimensions present in the pre/post dataset. Five factors were found, following Kaiser’s criterion (eigenvectors > 1), but only two of those were mirrored across the pre/post statement set, as required to allow us to test change (Supplemental Materials 2). Those that did emerge from the PCA were the statement pairs intended to test self-orientation (Self-E and Self-I in Table 2), which we averaged for scales (Rho 0.47 and 0.44 respectively for pre and post, both *p* < 0.000, Table 2). These are called Self-Pre and Self-Post.

**Table 3:** Statistics for all scales emerging from the pre/post surveys, including follow-up

Phenomenon	Pre (97)	Post (81)
Climax thinking	Climax-Pre (8)	Climax-Post (8)
	Alpha: 0.69	Alpha: 0.70
	Mean: 2.88	Mean: 2.72
	SD: 0.61	SD: 0.69
Self-orientation	Self-Pre (2)	Self-Post (2)
	Rho: 0.47***	Rho: 0.44***
	Mean: 3.56	Mean: 2.43
	SD: 0.91	SD: 1.05

Descriptions include scale names (number of statements), Cronbach’s alpha, mean and standard deviation

***p < 0.001

We carried out Spearman tests to see if our coastal or random treatment groups differed on these scales. Atlantic was positively associated with the Climax-Pre score (rho = 0.26, *p* < 0.01) and Northumberland was weakly negatively associated (rho = −0.24, *p* < 0.05); none are associated with any of the Climax-Post scores. Self-Pre was weakly negatively associated with the past priming treatment (rho = −0.22, *p* < 0.05), and Self-Post was weakly negatively associated with the Bay of Fundy coast (rho = −0.24, *p* < 0.05). No other correlations were significant of the 28 combinations run.

### Impact of the Focus Groups on Climax Thinking Measures

Simply participating in our focus groups decreased self-orientation when pre/post scores are compared (*p* < 0.001; Table 4). The treatments had very different outcomes, however. The control focus groups only slightly decreased in self-orientation (*p* < 0.05). The past priming had no significant impact on either scale, perhaps because of the small number of past-primed individuals who completed both the pre- and post-test (*n* = 13; Table 4). The future and altruism priming both significantly decreased self-orientation (*p* < 0.001). Climax thinking also decreased significantly under the future priming (*p* < 0.01), Table 4).

**Table 4:** T-tests between pre and post test means for each treatment group, for those who did both surveys, and between control and experimental treatments within each period

Treatment (n) and scale	Pre-test	T-test pre control-other (p)	Post-test	T-test pre-post (p)	T-test post control-other (p)
**All** (*n* = 77)					
Climax	2.89	–	2.71	1.71	–
Self-orientation	3.60	–	2.39	7.68***	–
**Control** (*n* = 22)					
Climax	2.85	–	2.91	−0.39	–
Self-orientation	3.41	–	2.55	2.81*	–
**Past** (*n* = 13)					
Climax	2.74	1.29 (0.21)	2.86	−0.52	0.20 (0.85)
Self-orientation	3.31	0.91 (0.37)	2.46	1.91	0.23 (0.82)
**Future** (*n* = 19)					
Climax	3.12	−0.88 (0.38)	2.49	3.47**	1.60 (0.12)
Self-orientation	3.87	−0.89 (0.38)	2.42	5.11***	0.06 (0.95)
**Altruism** (*n* = 21)					
Climax	2.88	−0.25 (0.80)	2.63	1.10	1.65 (0.11)
Self-orientation	3.90	−1.51 (0.14)	2.19	6.55***	1.22 (0.23)

*p < 0.05; **p < 0.01; ***p < 0.001

Recalling that these differences before and after the focus groups may be the result of the different statements used in the pre- and post-tests, we also compared the control measures of climax thinking and self-orientation with those for all three treatments within pre- and post-tests (Table 4). There are no statistically significant differences in the pre/post analyses, perhaps because of sample size issues. However, two of the three significant relationships identified using the pre- and post-tests hold up in the control treatment visual comparison: future priming decreases climax thinking and altruism priming decreases self-orientation. Altruism also seems as effective as future priming on climax thinking using this control treatment comparison (*p* = 0.11 compared to *p* = 0.12), though it was not significant in the pre/ post-tests.

### Impact of the Treatments on Conversations about Coastal Adaptation

Because focus group participants were effectively doing the priming for one another, rather than being presented with a uniform story by facilitators, the experiences of each treatment were not identical. Supplemental Materials 1 contains a detailed summary of what it was like to participate in each of the focus groups. Fundamentally, however, the focus groups were comparable in experience, so here we discuss the nature of the conversations about coastal adaptation and whether those differed by treatment. Responses to individual nature-based options did not differ by treatment and are discussed elsewhere (Sutton 2020).

The discourse around adapting to coastal changes indicated that most focus group participants did not yet feel the issue was urgent, but this did vary somewhat by treatment. Past-primed participants were quite apathetic about adaptation. A few participants in the past-primed focus groups talked about change as something that is inevitable and part of coastal living, saying that it was futile to have an opinion or be proactive. Some past-primed participants were concerned about the heritage and tourism implications of coastal changes they saw, but they seemed to think that everything possible to combat coastal change was already being done. A subset felt they should not be concerned because the major effects of climate change will not have an impact on them because of those established protections. For instance, a highly vulnerable male participant in the Bay of Fundy past-primed focus group said:

I know that if, you know, if the tide rises, well, not much we can do about it but if the tide rises a metre, I’d have 2 ft of water in my living room but, I have good faith in what we have so far, I think we’re protected… I also chose to live this close to the water, and it’s been here for, well, not my house but the property itself has been there for a long while. I’m hoping to get a, you know, a couple hundred years more out of it… Well, I can walk off my front deck, take three steps and jump in the Bay of Fundy. Also, on a real high tide, the tide is actually probably a little higher than my main floor, so I rely on the walls, the wall that I have out in front and all around property to protect it.

By contrast with those participants, others, particularly in the Northumberland past-primed focus group, were in fact concerned about the impacts of climate change and believed that changes need to be made. However, these participants felt that the next generation should be the group to champion those changes.

In the altruism-primed focus groups, participants seemed aware of the need to change and adapt and were mostly willing to make that change. Participants used language like “come together” when describing ways in which their communities have worked together towards a common goal, with examples about fighting wastewater treatment facilities and fish farms. There was a sense from altruism-primed participants that in moments of clear need people could be spurred into action. The ways in which participants referred to the nature-based approaches suggested that they were concerned about making responsible and positive changes as opposed to making changes simply to protect what they had. They made references to being optimistic about the potential to adapt to climate change with phrases like “if we could do it collectively,” referencing their belief that as a community they could make a difference and work towards a solution for coastal climate change. On the other hand, the majority of participants in the altruism-primed focus groups felt that for most people the threat of climate change was not imminent enough to “rally the troops” for a mostly unacknowledged and distant threat. The echoing of military language suggests that the priming may have been more relevant to participants than they realized.

The future-primed focus groups also featured quite urgent language around the need for change, with the majority of rationales echoing the treatment. A male Northumberland Strait participant said, “I think there’s an old saying that says, this is not our land, we don’t own it, we’re just borrowed it and we should leave it how we found it.” This quote is characteristic of many participants in the Northumberland Strait future-primed focus group, as they appeared to be conscious of the trajectory of coastal risk and the need for change. The Northumberland future-primed group conveyed an understanding of the urgency of adapting but aligned strongly against the idea of wasting time on measures that may not last (e.g., accommodation such as raising homes). Bay of Fundy future-primed participants did not necessarily see changes in the handling of climate change as urgent, but they did advocate working towards solving climate change for future generations, even if that resulted in major changes to their coast. The majority of participants in the first Bay of Fundy future-primed group agreed that we have to make changes so that future generations can live like the current generation does, with one female participant saying:

… in order to allow people to continue to live the way that we do… it’s going to be a huge expense but if we don’t make those adaptations and protect that infrastructure now, then that just means that in the future those communities are going to begin to disappear because people won’t be able to have a livelihood there… But if we don’t act now, we’re going to be faced with a much bigger problem in the future.

The Atlantic future-primed focus group participants were the most concerned with the need to adapt soon. One of the female participants in the group put it this way: “I definitely think we have to do something now because we can see the changes and they’re coming fast. And if we don’t do something now it’s going to be too late.” The commonly repeated reactions of Future-primed participants suggest that the treatment was able to emphasize the time-sensitivity of adapting to climate change.

We perceived very little urgency in our control groups, even though participants talked about major infrastructure, such as main roads, being impacted. Recall that the control groups received only facts about climate change, its impacts, and adaptation options. One exception around urgency was in our Northumberland Strait control group, where one individual felt it was already too late to be responding to coastal climate change and that we should start making changes immediately. Overall, the control groups did not appear interested in coastal adaptation, except to protect existing coastal infrastructure and shoreline locations.

## Discussion

This research used a novel climax thinking framework to design experimental focus groups to test the influence of priming treatments on perceptions of coastal adaptation among coastal residents in Nova Scotia, a coastally vulnerable jurisdiction. Participants were guided to provide the content of those priming discussions, ensuring the local relevance of the content. This guided approach meant the experiences of each treatment were not identical. The relative consistency of experience within each treatment, however, suggests this was a viable approach compared with using “canned” content from less authentic and trustworthy sources than fellow residents. The climax thinking measures and focus group discussions both suggest that we can reduce climax thinking—resisting landscape change based on a belief that the current landscape conditions are ideal, even fated, and deserve to persist—by using future- or altruism-focused communication strategies. This section explores the theoretical, methodological, and applied implications of this work.

### Measuring Climax Thinking

It is relatively easy to see, in retrospect, that while the content of the statements in the pre- and post-test are parallel, the sentiment and intensity may not be. For instance, the past-exceptionalism statement in the pre-test starts with “I feel grateful…” where the post-test analog has a more neutral tone. Similarly, the past-ignorance post-test statement starts with “It feels good…” and the pre-test is more neutrally phrased. So, there are reasons other than the treatment that the scores will vary between the pre-test and post-test. But the two statements composing each of the Self scales are strongly parallel in their content as well as sentiment, so we have greater confidence in this scale than we do in the Climax ones.

Despite the above, we argue for reasonable instrument validity given it is a first full operationalization (Chappell et al. 2020). Both statement sets appear to have face and content validity in relation to early descriptions of the concept (Sherren 2021). For criterion validity—how well findings with the instrument align with measures taken in other ways—we can compare test results with our qualitative analysis. We knew before the pre- and post-test data was provided to us that some priming approaches worked better than others. When we were confronted with inadequate recruitment to have a full replication of treatments on the Bay of Fundy coast, it was easy for us to choose the two treatments that we would replicate. The discussions that came after the priming treatments were substantively more productive and action-oriented in the future- and altruism-primed groups than in the control or past-primed groups, and this is consistent with our measurements. Construct validity is often assessed via correlation with measures known to vary in predictable ways with the phenomenon in question, but this framework is too novel for such hypothesized relationships, and our survey design is too limited to support such claims.

That said, this study suggests that climax thinking is likely not one thing, but several. Neither pre- nor post-tests were unidimensional in this operationalization. Factor analysis (Supplemental Materials 2) did suggest that the statement pairs for each given dimension (past, future, self, other) were found in the same factor in all but one case (statement 1a appeared in its own factor), but the causes (exceptionalism and ignorance) were not. This may suggest that individual dimensions of climax thinking (space and time) have more explanatory power than the drivers, and that they likely need to be individually pursued, perhaps drawing on extant theories such as construal level theory, social distance, or time perspective (Zaval et al. 2015).

### Influencing Climax Thinking

Of the three treatments, future priming worked best in focus group discussions, and this is supported by the tests (pre/post for both scales, control/treatment for climax). It is also the least novel among those tested (Stern et al. 2020). Future priming seemed to instill a sense of urgency for coastal adaptation the other two did not. It was also better received by participants than the altruism treatment, while having rather similar results. The future treatment may also have been more salient for our participants, the majority of whom self-identified as parents. These participants were more open to thinking about the future and making changes to prepare for the future they envisioned, even if they would not be the beneficiaries. This fits with Beaulieu et al. (2016) who find that asking participants to describe what they want the future to look like may increase support for action towards it. In asking participants to think about what they wanted future generations to experience or enjoy about the coast, intergenerational equity can be conceived of as a legacy passed on by the current generation for the well-being of those to come (Cooper and McKenna 2008; Hurlstone et al. 2020). The need to improve our capacity for considering the future in the present aligns with the work of those like Roman Krznaric (2020) and The Long Time Project (2020) who are working on building “long-termism” in the face of uncertainty. Priming in communication is only a starting point: a more systemic intervention or cultural shift is needed.

We used the “wartime mobilization” altruism treatment to develop a sense of shared challenge and to assign meaning to what might be seen as a local sacrifice—coastal adaptation. Our measures suggest this priming was effective in decreasing climax thinking and thus resistance to landscape change (pre/post for self; control/treatment for both) but the focus group discourse was more mixed: participants took longer to engage in the group discussion, suggesting that some participants may not have found the topic relatable. The wartime mobilization comparison was used to show that we can make large-scale changes in short periods, as is needed to properly address climate change, but Kester and Sovacool (2017) consider the comparison incongruent, as well as potentially unhelpful. Indeed, when asked at the end of the focus groups what they thought about the treatment they had received, a few of our participants used the term “apples and oranges” to dispute the congruence of climate and war. A recent review of similar “emergency” frames does indicate their utility in engaging and energizing mass publics but also points to the risk of emotional costs, such as fear and demotivation (Patterson et al. 2021). There may be better ways to inspire a sense of shared challenge and responsibility in the face of climate change, but our results suggest that this one may be worth further exploration.

Both the future and altruism treatments speak to the importance of building compassion and empathy. This aligns with the findings of scholars such as Katrina Brown et al. (2019), who note, “Whether empathy leads to sustainability depends on whether empathic responses transcend differences between groups within a place (e.g., ‘incomers’ vs. ‘natives’) and across spatial boundaries (proximate vs. distant)…” (p. 13). Brown et al. (2019) also provide useful methodological suggestions for exploring empathy. Fostering empathy with those who are more geographically or socially distant (as opposed to future generations) can be difficult, but researchers who work with relatively similar groups and encourage “perspective taking” of the other find that it leads to more sustainable decision-making (Ortiz-Riomalo et al. 2021). Recognizing that not everyone has the same capacity to adapt as our older, wealthier participants did is an important part of garnering acceptance and support for shared climate adaptation; for instance, Nova Scotia has an income gap between rural and urban areas, and those who live in rural areas have a greater chance of living in an older or poorly maintained home (Manuel et al. 2015). Future researchers should explore authentic and equitable ways of generating and sustaining empathy for “others,” and the practical limits of doing so.

Our past-oriented priming treatment was counterproductive based on all the data we collected. Participants in the past-primed focus groups largely rejected the nature-based options presented, citing the expense of the approaches—“who will pay?”—and uncertainty about their reliability as reasons to defend their current coastal infrastructure. Past-primed participants expected the government to limit their need for change and cover any costs when they did. This may not be all negative for climate discourse involving such people; Davydova et al. (2018) found that there is a greater sense of collective control (belief that something can be done collectively to mitigate a threat) and perception of a threat from climate change when emphasis is placed on the responsibility of government in both contributing to and mitigating climate change effects. However, for our purposes the past treatment may have encouraged an aversion to change and adaptation by reminding people of what they have already lost on the coast; in other words, it is possible that past-priming reinforced the *status quo* bias that we were hoping to challenge. Morton et al. (2011) argue that focusing on losses will increase uncertainty and decrease an individual’s likelihood to engage in pro-environmental behaviors. Our past-primed focus group participants expected to sustain current landscape uses and maintain their coastal heritage intact despite the challenges presented by climate change.

## Conclusion

Understanding how to speak to people affected by coastal climate change about the difficult decisions ahead is critical to adaptation. In rural places, the cost of ongoing coastal defense is becoming increasingly impractical, and approaches such as living shorelines and managed retreat are being discussed. We tested the impact of three experimental priming treatments inspired by climax thinking on discussions of coastal adaptation in 14 online focus groups. Integrating results from mixed methods, the most productive discussions will orient people toward considering the needs of future generations or, perhaps more riskily, explore the meaning that can be found in sacrifice and altruism. By contrast, highlighting past changes is likely to increase resistance to adaptation rather than decrease it. Climax thinking provided some novel leverage points for the priming experiments, but it is not a unidimensional concept for measurement purposes.

Our study is also not a representative sample of Nova Scotians, due in part to the requirement that participants be homeowners and the phone recruitment used. Likewise, as our participants predominately identified as middle- to upper-class individuals, they are not representative of rural Nova Scotia or the most vulnerable coastal populations. A more representative perspective could be acquired via population-level surveys though it would be difficult to replicate the focus group priming experience that way. Our focus groups were held online pre-Covid, when that mode was unfamiliar to many, and this undoubtedly had an impact on their experience: we would expect the post-pandemic population to interact even more naturally in that mode than our participants did. Finally, the duration that such priming effects might last is also unclear and begs a longitudinal design.

## Supplementary Information

Coast climax OR 1

Coast climax OR 2
