# Supplementary material for: Climax thinking on the coast: a focus group priming experiment with coastal property owners about climate adaptation
Source: Environ Manage. 2022 Jun 30;70(3):475–88. doi: 10.1007/s00267-022-01676-x (PMC9381476; doi:10.1007/s00267-022-01676-x)
Supplement: Supplementary file 1 — Coast climax OR 1 [file 267_2022_1676_MOESM1_ESM.pdf]

Sherren K, Sutton K, Chappell E. (2022) Climax thinking on the coast: a focus group priming experiment with coastal property owners about climate adaptation. *Environmental Management*. DOI: 10.1007/s00267-022-01676-x

Supplementary materials 1

Here we describe the results of the priming questions as participants answered them, as a way of understanding the experiences participants had and how consistent each priming treatment was between focus groups that used them.

Past priming: coastal change trajectory

Within the past priming focus groups, participants were typically initially a little confused by our question, "have there been any changes to buildings or infrastructure for any reason in the time you have lived on the coast?". Once we clarified that we were looking for them to identify changes other than those related to climate change, dialogue flowed better. Participants identified shifts in both population demographics and in seasonal population due to tourism. For instance, participants felt more "come from aways", that is, people not from the area or from Nova Scotia, and older individuals were moving in even as local youth were leaving in larger numbers than before. They also noted a change in industry and the economy in their communities. For some participants, abandoned wharves and buildings were a sign that their communities had migrated away from the fishing industry to another economic source though not necessarily a more profitable one. One participant mentioned the fact that traffic used to flow through their small town on the main road until traffic was redirected when the highway was built 16 years ago, affecting the stores and travel to the area. When asked if their own reasons for living on the coast had changed since they moved to the coast, none of the participants felt that their reasons had changed.

When asked about the importance of preserving the existing infrastructure and buildings on their coastline four main themes emerged (Table S1.1). Some participants felt that it was important for things to remain where they are for economic, cultural, and/or social reasons. A fourth theme of resignation or futility appeared as a contradiction to the other three. The economic reasons were that participants felt their communities rely on fishing infrastructure and wharves, coastal buildings and shops for tourism, and waterfront development to bring in business and encourage tourism growth. Culturally, participants were against moving existing coastal infrastructure because the buildings and houses are a large part of what Nova Scotia is known for and proud of. As one female participant on the Atlantic coast put it:

> I think Nova Scotia is you know hallowed ground. It's supposed to be the Ocean Playground [once written on NS license plates] and we don't want it the Ocean Wasteland and I think it's a big part of tourism and it's pretty along the coastlines and I think it would be terrible if we lost that… it's extremely important to generations too.

Table S1.1. Participant identified themes for the importance of preserving existing infrastructure and buildings on their coastline

Themes:	Important			Not Important
	Economics	Cultural	Social	Resignation/Futility
How important is it to preserve the existing buildings and infrastructure along your coastline?	Fishing infrastructure and wharves are livelihood	Buildings and houses are the pride of Nova Scotia	Provides a sense of community	Waste of money to preserve infrastructure
	Buildings bring in tourism	Moving heritage houses would impact families	Coastal infrastructure defines the community	Losing battle with nature
	Waterfront development attracts tourism	Houses on coast tell history of NS	Community history	Coast is always changing

Others were opposed to the idea of moving houses that have been there for generations due to the negative impacts it would have on the families that live there. The knowledge that some of the houses on the coast are very old and tell a part of Nova Scotia's history also made some of them feel like it is important to preserve the coastal infrastructure as it is. The social aspect of maintaining the infrastructure where it stands is that it brings with it a sense of community. Many participants felt that the coastal infrastructure defines their community and tells the history of the community:

> …you know a lot of them are what you grew up with however I guess times change and it's somewhat important but I guess you know times do change and evolve and so that's kind of what you have to live with sometimes, that's the reality here, but — But, it'd be good to see some of those historic houses and that still remain. If you could find a use for them… Like any community it defines; this place goes here, you know. I can trace my ancestry back almost 250 [years] here so you're going back a long, long time, and your ancestors built this place up and now you see it kind of deteriorating and regressing in some ways so that's kind of sad. (Male Bay of Fundy participant)

Such responses were not uncommon among participants and closely ties the social importance of infrastructure on the coast to the cultural aspects. It is indicative of the predominant attitude within the past priming towards maintaining the coastal landscape.

A very small subset of participants did not oppose moving existing buildings as they felt that it was either naïve to believe things will stay where they are or a waste of money to try and keep things where they are because the coast is always changing and fighting nature is a losing battle:

> …I don't think that we should be putting money- or government money in particular into trying to save buildings from natural forces that we will never be able to defeat. In the case of infrastructure, a road might be changed to a bridge for example, if there was a significant amount of erosion, but in terms of buildings, if it's not sustainable for a building to be in an area then you know I would have a hard time saying well we should be sinking a lot of money into keeping that building there when there's so many other places where we should be putting money. And you can't fight nature, Nature's coming down on you, you need to get out of the way. (Female Atlantic Ocean participant)

Future priming: looking towards a future coastline

In the future priming, when we asked, “what aspects of living on the coast do you hope future residents will be able to experience?”, participants needed to be reminded that we were not looking for them to think about future residents in terms of the potential effects of climate change as we had previously described to them, but just based on the aspects of coastal living they enjoyed as they do now. When asked what they enjoyed or hoped future residents will enjoy about the coast, responses could be broken down into three categories: atmosphere, activities, and belonging. For atmosphere, participants talked about the sights and sounds, the breeze, and the general ambiance on the coast as something they hoped future generations would also get to enjoy. Activities consisted of hiking, walking on the beach, gardening, fishing, clamming, and other marine activities. These activities were things that participants enjoyed doing themselves but were also things that they considered to be part of the coastal living experience:

> … the quality of life of living in a small community, just being able to- the cleanness of the air and the sound of the waves and just being able to get out and walk on the beach. And I think it's just a quality of life but not, it's not for everyone but for people who enjoy it, it's priceless. So, I think there is a certain connection there with people who have been in a certain area or their families have been there for many, many years. And just the contentment that you know of living in a place where you just feel you belong (Female Bay of Fundy participant).

Participants often described that they would like the sense of belonging and community connectedness that comes with living on the coast to continue to exist for future residents.

As we moved participants along in this priming section, we wanted to know if participants thought their current coastal adaptation would work for future residents. In this, participants were unsure; many felt that over time their current coastal adaptation would cost too much money to keep up with climate change. This was connected to expressions that climate change is something that we cannot stop, only manage. There was also a sense from some participants that currently employed adaptation approaches were short-lived and would only delay coastal changes.

In this treatment a brief poll was implemented, asking: “Which is more important? Extending the life of current coastal protections and infrastructure locations for current residents, even if it increases vulnerability for future residents or adapting coastal protection and infrastructure now to reduce the vulnerability of future residents, even if it means change for current

residents?". In the subsequent discussion many participants voiced skepticism that there could be effective coastal adaptation though they were somewhat hopeful a solution could be found. As a male participant in the second Bay of Fundy focus group explained:

> I said adapting for the future [was more important in the poll]. I mean it's kind of a hard question really but a lot of what we do seems to be only temporary, you know with the hard walls and all the stuff we were discussing before. And you know if we could somehow slow it down in the future you know I think that would be the best option.

Nevertheless, the majority believed that we do in fact need to adapt now to extend coastal living for the future, with a smaller number going even further than that, stating that if we do not start adapting for the future now it will be too late. But there were other minority opinions. A few participants were of the mind that we should focus on current residents, but they were generally agreeable with the idea that future generations should be considered as well. There was also a subset of participants who believed that it is expensive to try and stop coastal climate change and as such there is no point in talking about adapting. Another small group of participants believed that we already should have started to adapt for the future (i.e., it is too late).

Altruism priming: finding meaning in collective sacrifices

Unlike the other priming treatments, this priming did not speak to landscape changes; instead, the priming asked participants to consider the kinds of sacrifices their communities had made in the past for the greater good and think about whether the same could be said about the present. When prompted to talk about the kinds of war efforts and sacrifices their communities made during World War I and II, participants across all altruism-primed focus groups initially hesitated before talking about their knowledge that many in their communities served in the war, did whatever needed to be done in their communities, rationed food and cared for others, worked in the coal mines, made supplies, fed people, or built homes for shipbuilders as their contribution to the war efforts.

When asked, "What made it possible for that kind of collaboration?", participants intimated that their communities were able to make these sacrifices because they were small communities that had a strong bond. The majority also said it was because everyone in the community knew of someone who fought overseas. Many felt that there was a different mindset in communities during the World Wars, unlike today, which made it more automatic for people to collaborate and help their neighbours out of self-reliance rather than a reliance on the government. These participants likewise felt that the way people collaborated then was because the wars were a threat to life and country, and a call to arms greater than anything we have now.

Such responses presaged our subsequent question, "Do you think we would be capable of accepting similar sacrifices and changes today?", with which a large majority of participants said yes. Their reasoning was that it is human nature to make sacrifices for others and that their communities are "all for one" communities with many examples of coming together to support individuals who are sick or in need, fundraising even by those who don't have much to give, and volunteering for those in the community who need help. A minority felt that it is not possible

for people to make sacrifices like they did for the World Wars if it was for something they cannot relate to. These participants gave examples of events happening on the other side of the world, people who “come from away” and move into their communities but choose not to get involved in community events, and the fact that in the media non-tragedies do not get the same sort of action that tragedies do. These answers suggest that it is where an ongoing sense of altruism and connectedness in these communities exists that also does a willingness to make sacrifices and changes for a good cause.

Another prompt asked, “Imagine one person’s or company’s property or infrastructure worsened coastal erosion for their neighbours. Would you expect to respond as a community in that case?”. Overall, participants felt that they would respond to coastal erosion on one person’s property as a community, especially if that person was not responsible for creating the problem. A female participant from the first Bay of Fundy focus group demonstrated, however, that you cannot rely on just your neighbours for support:

> I think that if it was affecting your property or something and the person that put it in didn't seem to be all that interested in doing something about the problem and that yes, I think others would come to your assistance. But again, it would be a matter of if the community is not going to be able to do anything for you because in a case like that what can they do really? It would just be a matter of getting through to your MLAs or whoever is in charge of your roads, the shoreline stuff, stuff like that anyways, getting hold of them and telling them what the problem is.

Others, like this male participant on the Atlantic coast, indicated that depending on the situation they would respond as a community. For these participants responding as a community was also contingent on whether the individual causing the worsening erosion took responsibility for their actions and made changes to rectify the problem:

> … I think it depends on the situation. I think if an individual is doing something to purposefully and wilfully – that is affecting the coastline – I think people are going to be not hesitant in the time of his need, like if something does happen people are still going to pitch in and help and try to get him back on his feet. But they're going to expect him to make changes to what he's doing, not continue on the way that he is. I think they'll still pitch in.

Even as our participants were mostly willing to respond as a community to coastal erosion on one’s property, they made it clear that there is an expectation of taking responsibility and ownership when it comes to the source of the problem.

When asked whose responsibility it is to pay for changes to coastal protection and infrastructure due to sea-level rise, the predominant response was that the responsibility lies with all levels of government. Participants emphasized the fact that in some cases municipal governments must rely on provincial and federal governments for the funds to adapt to coastal changes. There was a consensus among participants that we ought to have included the private sector and companies as a group they could have chosen. They felt that those in the private sector that have a hand in adding to climate change should be responsible for dealing with the coastal changes that result. For example, a male participant on the Northumberland group said:

> Well, they [companies] can be [responsible], yes. It is the case in our area that's for sure. And I know in the south shore mining companies and so forth are causing quite a few problems, so yes, if they are causing the issue, whether it's land-based or whatever they should be part of the solution as well.

There were those who believed that it should be a group effort including individuals from all suggested groups: government, NGOs, affected residents, community groups, and owners of future builds. They felt that in some cases the responsibility starts with the government allowing people to build in at-risk areas and those people not taking precautions on the coast. As coastal changes affect both public and private property their belief was that involving everyone and holding them responsible would help in finding a solution that works.

The final question we asked altruism-primed participants was about how coastal residents could take responsibility for responding to coastal changes. Participants suggested that coastal residents "spark awareness" of the effects of coastal changes in their communities and pay attention to the changes. They also felt that by avoiding building in problematic areas residents could take responsibility.

Consistency of priming and coasts

Allowing the focus group participants to shape the priming in response to prompts means that, as researchers, we lose control of consistency. We acquire authenticity, but at what cost? The stories elicited by the facilitated priming varied somewhat across instances with some coasts focusing on different parts of the discourse and having different reasons for their responses. Within the past priming, for instance, the Northumberland group stands out from the other two coasts in that the stories elicited from the priming were different. Participants in this focus group were very concerned about the tourism and economic effects of coastal adaptation rather than the effects of climate change, which is perhaps caused by the relatively recent subdivision of coastal farm lots there for cottage use. From the tone of the focus group one could sense that they were not very interested in adapting to climate change before they had to. Apart from the focus on tourism and economics in the Northumberland group, the past-primed focus groups all told much the same kinds of stories.

There were a few differences in the future priming, with the second Bay of Fundy future-primed focus group being more concerned with how different the coast would look if it was adapted for the future generation and less concerned about impact on future generations as a whole. Despite that, they still felt that adapting to coastal changes is something owed to future generations. There was one prominent participant in this Bay of Fundy group who disagreed with the others because he was thinking about how it would directly affect his children and grandchildren:

> You know, a lot of people and I think that's what they're doing, they're saying 'hey well you know what I'm 60 years old, 50-60, it ain't going to affect me in 20 years, I won't be here, so. A lot of people have different thoughts but for me you know I have children I'd like to see them enjoy the ocean, the coast and you know have a safe place to live in the future so I'm optimistic that hopefully we can find something. And we should try instead of, you know, not trying, so that's my opinion.

The first Bay of Fundy future focus group was more concerned about future generations and how they would support themselves if they stayed, citing many generations of living on the coast and feeling like it was important to be able to pass that on. Conversation within the priming for this group was influenced by two of the participants recognizing each other's voices and telling stories from their own community. By contrast, the Northumberland and Atlantic future focus groups had very similar discourse throughout the priming section. This would suggest that the future priming was successful in eliciting similar perspectives across coasts.

In the altruism priming, stories were consistent between focus groups as participants shared stories of how their communities had come together in war efforts and gave similar examples for how they come together now. There were differences in how they thought their community should react today, with a strong justice and responsibility story appearing in all groups but quite prominently in the first Bay of Fundy focus group and the Atlantic focus group, while a sense of community/collaborative response still appeared in the other two focus groups. The Northumberland and Atlantic Focus groups believed the wartime mobilization priming to be "apples to oranges" (this was a response elicited outside of the 'treatment' section when we asked people what they thought about the priming) in comparison with climate change while the first Bay of Fundy group found aspects of it to be useful. Despite these differences in opinion about the altruism priming, most participants understood the priming and responded positively to it.
