# Supplementary material for: Climax thinking on the coast: a focus group priming experiment with coastal property owners about climate adaptation
Source: Environ Manage. 2022 Jun 30;70(3):475–88. doi: 10.1007/s00267-022-01676-x (PMC9381476; doi:10.1007/s00267-022-01676-x)
Supplement: Supplementary file 2 — Coast climax OR 2 [file 267_2022_1676_MOESM2_ESM.pdf]

Sherren K, Sutton K, Chappell E. (2022) Climax thinking on the coast: a focus group priming experiment with coastal property owners about climate adaptation. *Environmental Management*. DOI: 10.1007/s00267-022-01676-x

Supplementary Materials 2

Table S2.1 Exploratory Factor Analysis results of all 16 pre- and post-test statements

Variable	Factor 1	Factor 2	Factor 3	Factor 4	Factor 5	Uniqueness
Q1A	0.1747	0.0637	0.1619	0.0245	**0.8020**	0.2954
Q1B	**0.7473**	-0.1430	0.0009	0.3311	0.2008	0.2712
Q1C	**0.5834**	0.1016	0.2300	0.0123	0.1301	0.5793
Q1D	**0.6533**	0.1710	-0.1223	-0.0255	0.4255	0.3472
Q1E	0.0866	-0.0883	**0.8306**	0.1229	0.0835	0.2727
Q1F	0.0321	0.0695	**0.8584**	-0.0604	0.0392	0.2520
Q1G	**0.7145**	0.2681	0.0710	-0.3186	-0.0341	0.3098
Q1H	**0.8254**	-0.0880	0.0750	0.0758	-0.0754	0.2939
Q2A	-0.0938	0.0911	0.3647	**0.6608**	-0.0532	0.4104
Q2B	0.1390	0.2122	-0.0516	**0.6653**	0.2425	0.4315
Q2C	0.1106	0.1349	-0.0758	**0.6244**	-0.2075	0.5309
Q2D	0.0689	0.1951	-0.3565	**0.4193**	-0.3161	0.5544
Q2E	-0.0238	**0.8229**	-0.0659	0.0024	0.1513	0.2950
Q2F	0.0685	**0.7770**	0.0255	0.1774	0.0053	0.3594
Q2G	-0.0284	**0.5359**	0.0363	0.2303	-0.0239	0.6571
Q2H	0.0784	**0.6260**	0.0431	0.0993	-0.4864	0.3537

Table S2.2 Eigenvalues for the exploratory factor analysis (S1.1)

Factor	Eigenvalue
1	3.07
2	2.47
3	1.76
4	1.41
5	1.07

Table S2.3 Factor scales for the exploratory factor analysis (S1.1)

Factor	Questions	Alpha	Mean (SD)
1	1B, 1C, 1D, 1G, 1H	0.75	2.40 (0.77)
2	2E, 2F, 2G, 2H	0.71	2.39 (0.88)
3	1E, 1F	0.65	3.56 (0.91)
4	2A, 2B, 2C, 2D	0.52	3.06 (0.78)

a) 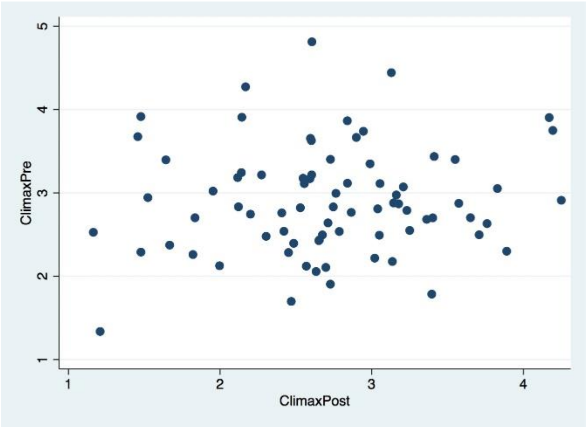

b) 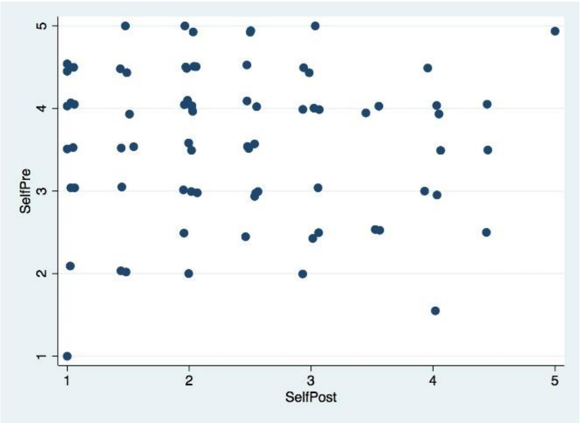

Figure S2.1 Scatterplots (with jiggle) of scales, a) Climax-Pre versus Climax-Post, and b) Self-Pre versus Self-Post
